# Recombinant BaMtx Preserves the Biological Properties of a Lys49 Phospholipase A_2_ from *Bothrops atrox* and Reveals Differential Responses in Tumor and Non-Tumor Cell Models

**DOI:** 10.3390/toxins18080338

**Published:** 2026-08-02

**Authors:** Daniel Torrejón, Angie Regalado, Alex Proleón, Víctor Otárola, Fanny Lazo, Edith Rodríguez, Erasmo Colona-Vallejos, Libertad Alzamora-Gonzales, Jherson Cisneros-Gutierrez, Jacquelyne Zarria-Romero, Miryam Paola Alvarez Flores, Renata Nascimento Gomes, Thatiana Corrêa de Melo, Ronnie G. Gavilan, Javier Cárdenas Tenorio, Félix A. Urra, Dan E. Vivas-Ruiz, Armando Yarlequé

**Affiliations:** 1 Grupo de Investigación Toxinas de Origen Animal y sus Antivenenos (TOXIVEN), Laboratorio de Biología Molecular, Facultad de Ciencias Biológicas, Universidad Nacional Mayor de San Marcos, Av. Venezuela Cdra. 34 S/N, Ciudad Universitaria, Lima 15081, Peru; daniel.torrejon@unmsm.edu.pe (D.T.); angie.regalado@unmsm.edu.pe (A.R.); victor.otarola@unmsm.edu.pe (V.O.); flazom@unmsm.edu.pe (F.L.); erodriguezq@unmsm.edu.pe (E.R.); ayarlequec@unmsm.edu.pe (A.Y.); 2 MODULANS Research Group, Faculty of Biological Sciences, Universidad Nacional Mayor de San Marcos, Lima 15081, Peru; ecolonav@unmsm.edu.pe (E.C.-V.); lalzamorag@unmsm.edu.pe (L.A.-G.); jherson.cisneros@unmsm.edu.pe (J.C.-G.); 3 Grupo de Investigación de Reproducción, Biología del Desarrollo y Ecotoxicología (BIOTOXIC), Facultad de Ciencias Biológicas, Universidad Nacional Mayor de San Marcos, Av. Venezuela Cdra 34 S/N, Ciudad Universitaria, Lima 15081, Peru; 4 Centre of Excellence in New Target Discovery (CENTD), Butantan Institute, São Paulo 05503-900, Brazil; miryam.flores@butantan.gov.br (M.P.A.F.); renata.gomes@fundacaobutantan.org.br (R.N.G.); thatiana.melo@butantan.gov.br (T.C.d.M.); 5 Centro Nacional de Salud Pública, Instituto Nacional de Salud-Perú, Lima 15072, Peru; ronniegavilan@gmail.com; 6 Laboratorio de Bioquímica, Facultad de Ciencias de la Salud, Universidad Nacional del Callao, Av. Juan Pablo ΙΙ 306, Callao 07011, Peru; jjcardenast@unac.edu.pe; 7 Laboratorio de Plasticidad Metabólica y Bioenergética, Núcleo Interdisciplinario de Farmacología e Inmunología, Instituto de Ciencias Biomédicas, Facultad de Medicina, Universidad de Chile, Santiago 8380453, Chile; felixurraf@u.uchile.cl

**Keywords:** *Bothrops atrox*, venom, Lys49 PLA_2_ homologue, *Pichia pastoris*, cancer cell lines

## Abstract

Lys49 phospholipase A2 (Lys49-PLA_2_) homologues are catalytically inactive snake venom toxins with diverse biological activities, but their functional characterization requires recombinant production strategies to overcome the limitations inherent to native venom-derived proteins. Here, we produced recombinant BaMtx (rBaMtx), a Lys49-PLA_2_ homologue from *Bothrops atrox*, in the *Pichia pastoris* KM71 expression system and evaluated whether its biochemical and biological properties were preserved. rBaMtx was secreted into the culture medium, purified by cation-exchange chromatography, and characterized by SDS-PAGE, Western blotting, RP-HPLC (85.9% purity), and MALDI-TOF mass spectrometry, confirming a molecular mass of 13,821 Da. Despite lacking PLA_2_ activity, rBaMtx retained pronounced myotoxicity in vivo, inducing dose-dependent plasma creatine kinase release and skeletal muscle damage comparable to or greater than native BaMtx. In vitro, rBaMtx reduced viability of murine 4T1 breast carcinoma cells (IC_50_ = 60.94 µg/mL), altered cell morphology, modestly increased IL-1β release, and produced context-dependent effects in paired human tumor and non-tumor cell models, differing from native BaMtx and crude venom. These findings establish rBaMtx as a reproducible model for investigating the mechanisms and biomedical potential of catalytically inactive Lys49-PLA_2_ homologues.

## 1. Introduction

Snake venoms are a repertory of bioactive molecules that have evolved to target essential physiological processes with remarkable potency and specificity. This molecular diversity has made snake venoms an important source of pharmacological tools and lead compounds for the development of therapies against cardiovascular, inflammatory, infectious, neurodegenerative, and neoplastic diseases [1]. Currently, eleven toxin-based drugs have been approved for human use, including six derived from snake venoms [2], underscoring their translational potential and supporting the continued exploration of venom toxins as scaffolds for drug discovery.

Several snake venom proteins and peptides have attracted interest as therapeutic leads due to their ability to modulate biological processes involved in cardiovascular diseases, pain, diabetes, and cancer [3]. In oncology, snake venom metalloproteases (SVMPs), snake venom serine proteases (SVSPs), and phospholipases A_2_ (PLA_2_s) have emerged as promising candidates due to their cytotoxic and anti-migratory properties [4]. Among these, PLA_2_s comprise catalytically active Asp49 enzymes and catalytically inactive Lys49 homologues [5]. While Asp49 isoforms hydrolyze membrane phospholipids in a Ca^2+^-dependent manner, Lys49 homologues retain potent membrane-disrupting activity through their cationic and hydrophobic C-terminal region despite lacking enzymatic function. This structural and functional diversity has made PLA_2_s attractive candidates for therapeutic development [6].

Several Lys49 homologues exhibit membrane-disrupting, cytotoxic, and cell-modulating activities, highlighting their therapeutic potential [7]. In this context, our group previously characterized BaMtx, a basic Lys49 PLA_2_ homologue isolated from *Bothrops atrox* venom, as a non-catalytic 13.8 kDa myotoxin capable of inducing edema and anticoagulant effects [8]. We subsequently demonstrated that BaMtx reduced the viability of MCF-7 cells and strongly inhibited the migration of the highly aggressive MDA-MB-231 breast cancer cell line, while decreasing NADH levels and mitochondrial oxygen consumption. These findings suggest that BaMtx modulates cellular processes beyond membrane disruption and support its further development as a promising anticancer toxin.

Despite these promising biological properties, further investigation of BaMtx is limited by the scarce availability and inherent variability of native venom-derived protein [9]. Recombinant expression has emerged as an effective strategy to overcome these limitations, enabling the production of homogeneous proteins suitable for structure–function studies and translational venomics research [10]. Recombinant toxins have also demonstrated their value as biologically active molecules, facilitating the exploration and expansion of the therapeutic potential of PLA_2_s and other venom-derived proteins [11,12,13].

Snake venom Lys49-PLA_2_s are compact proteins stabilized by multiple conserved disulfide bridges [7], a structural feature that poses important challenges for their heterologous production. Although recombinant expression in *Escherichia coli* has been successfully achieved for several venom PLA_2_s, these proteins are frequently recovered as insoluble inclusion bodies, requiring denaturation and refolding procedures to recover biologically active molecules [14]. In contrast, the methylotrophic yeast *Pichia pastoris* has emerged as a highly successful platform for heterologous protein production because of its ability to express foreign proteins at high levels, perform eukaryotic post-translational modifications, including oxidative folding and disulfide bond formation, and efficiently secrete recombinant proteins into the culture medium [15]. Moreover, the low secretion of endogenous proteins by *P. pastoris* facilitates the recovery of heterologous products with high purity and preserved structural integrity, making this system particularly attractive for the production of structurally constrained venom toxins and for investigating their structure–function relationships and biomedical potential [10,16,17].

Therefore, this study reports the heterologous expression, purification, and characterization of BaMtx in *P. pastoris* (rBaMtx). The recombinant protein was biochemically and functionally compared with its native counterpart, and its antitumor activity was evaluated in murine and human carcinoma models, including 4T1 breast carcinoma, A549 lung carcinoma, and SK-MEL-28 melanoma cells, using tissue-matched non-tumorigenic cells as controls. These findings establish rBaMtx as a functional recombinant Lys49-PLA_2_ and provide a scalable platform for future structure–function and translational studies.

## 2. Results

### 2.1. Expression, Purification, and Biochemical Characterization of rBaMtx

**Figure 1:**
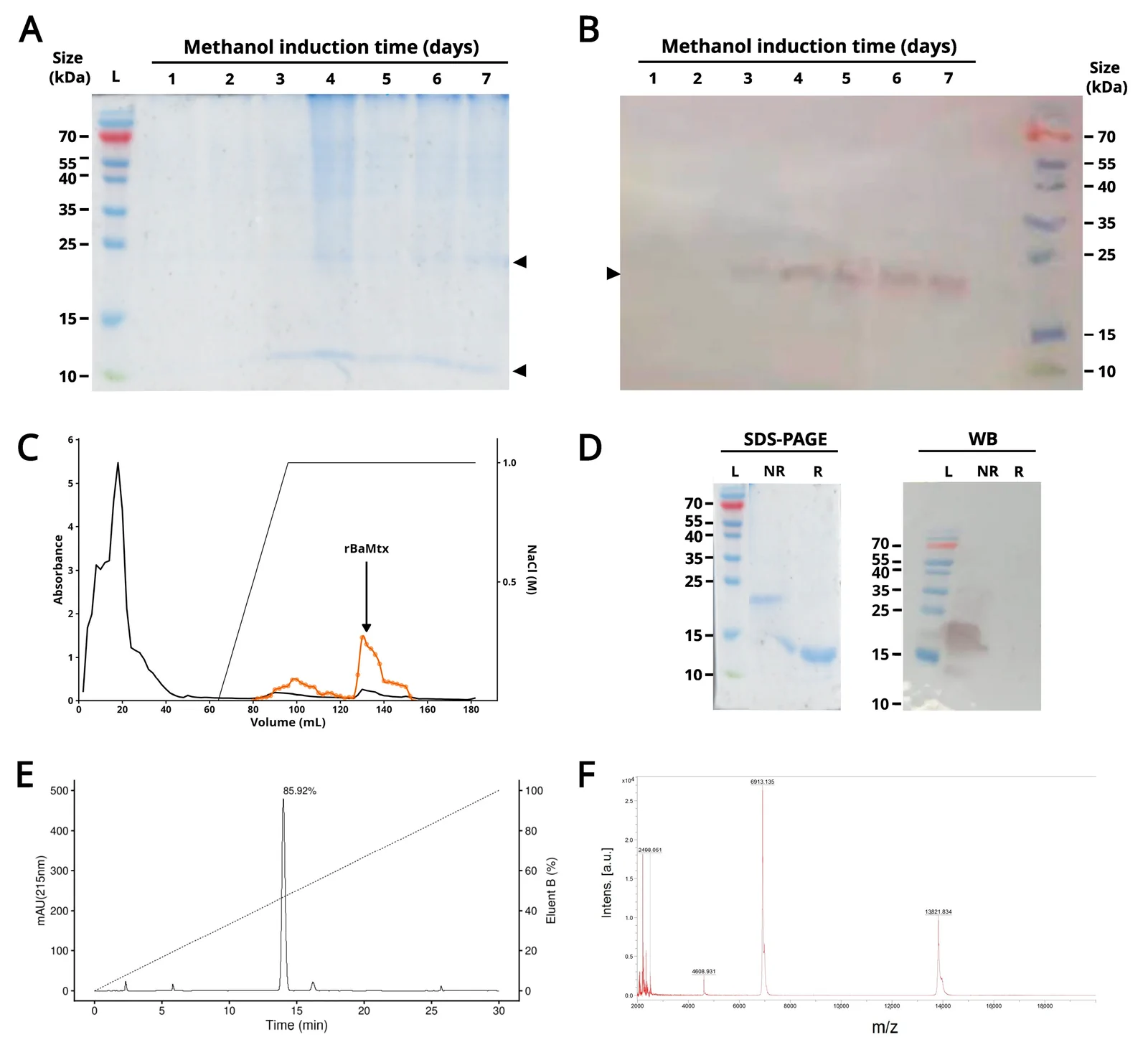
Expression, purification, and characterization of rBaMtx. (**A**) SDS-PAGE analysis of the culture supernatant (secreted protein profile) during rBaMtx expression *in P. pastoris* over 1–7 days of methanol induction. L, molecular weight marker; arrowheads indicate the bands corresponding to rBaMtx. (**B**) Western blot analysis of the corresponding culture supernatants collected during methanol induction; the arrowhead indicates the band corresponding to rBaMtx. (**C**) Cation-exchange chromatographic purification of rBaMtx. The black line represents UV absorbance (280 nm, left axis) and the orange line represents the NaCl gradient (right axis, M); the arrow indicates the fraction selected for further analyses. (**D**) SDS-PAGE and Western blot analyses of purified rBaMtx under non-reducing (NR) and reducing (R) conditions. L, molecular weight marker. (**E**) RP-HPLC profile of purified rBaMtx showing a major peak corresponding to 85.92% purity; the dashed line represents the acetonitrile (eluent B) gradient (right axis, %). (**F**) MALDI-TOF mass spectrum of purified rBaMtx, showing the singly charged ion [M+H]^+^ at m/z [insert value] corresponding to the expected molecular mass of rBaMtx.

The BaMtx coding sequence was successfully cloned into the pPIC9 expression vector, and the integrity of the recombinant construct was confirmed by nanopore sequencing (Supplementary Figure S1). Positive *P. pastoris* KM71 transformants were identified by colony PCR and selected for heterologous expression (Supplementary Figure S2). Methanol induction of positive transformants resulted in the extracellular secretion of rBaMtx. Two faint protein bands of approximately 12 and 23 kDa were detected by SDS-PAGE. The ~23 kDa species was specifically recognized by polyclonal anti-BaMtx antibodies, with the strongest signal observed after 4–7 days of methanol induction (Figure 1A,B).

The chromatographic elution profile and purity assessment of rBaMtx are shown in Supplementary Figure S3A. Purification of rBaMtx by cation-exchange chromatography, monitored by ELISA, yielded a distinct immunoreactive peak eluting during the 1.0 M NaCl isocratic phase (Figure 1C). SDS-PAGE analysis of purified rBaMtx under non-reducing conditions revealed a predominant band at ~23 kDa together with a faint band at ~13 kDa (Figure 1A). Under reducing conditions, the higher-molecular-weight band disappeared, whereas the ~13 kDa band became predominant. Western blot analysis using polyclonal anti-BaMtx antibodies detected immunoreactivity only under non-reducing conditions, corresponding to the ~23 kDa species (Figure 1D).

Analytical RP-HPLC revealed a predominant rBaMtx peak with an estimated purity of 85.92% (Figure 1E). MALDI-TOF mass spectrometry revealed a major peak at m/z 13,821.6 (Figure 1F). Catalytic PLA_2_ activity assays showed no detectable enzymatic activity for rBaMtx, whereas native BaMtx exhibited low substrate hydrolysis (Supplementary Figure S3B).

### 2.2. In Vivo Myotoxic Activity and Morphological Analysis

**Figure 2:**
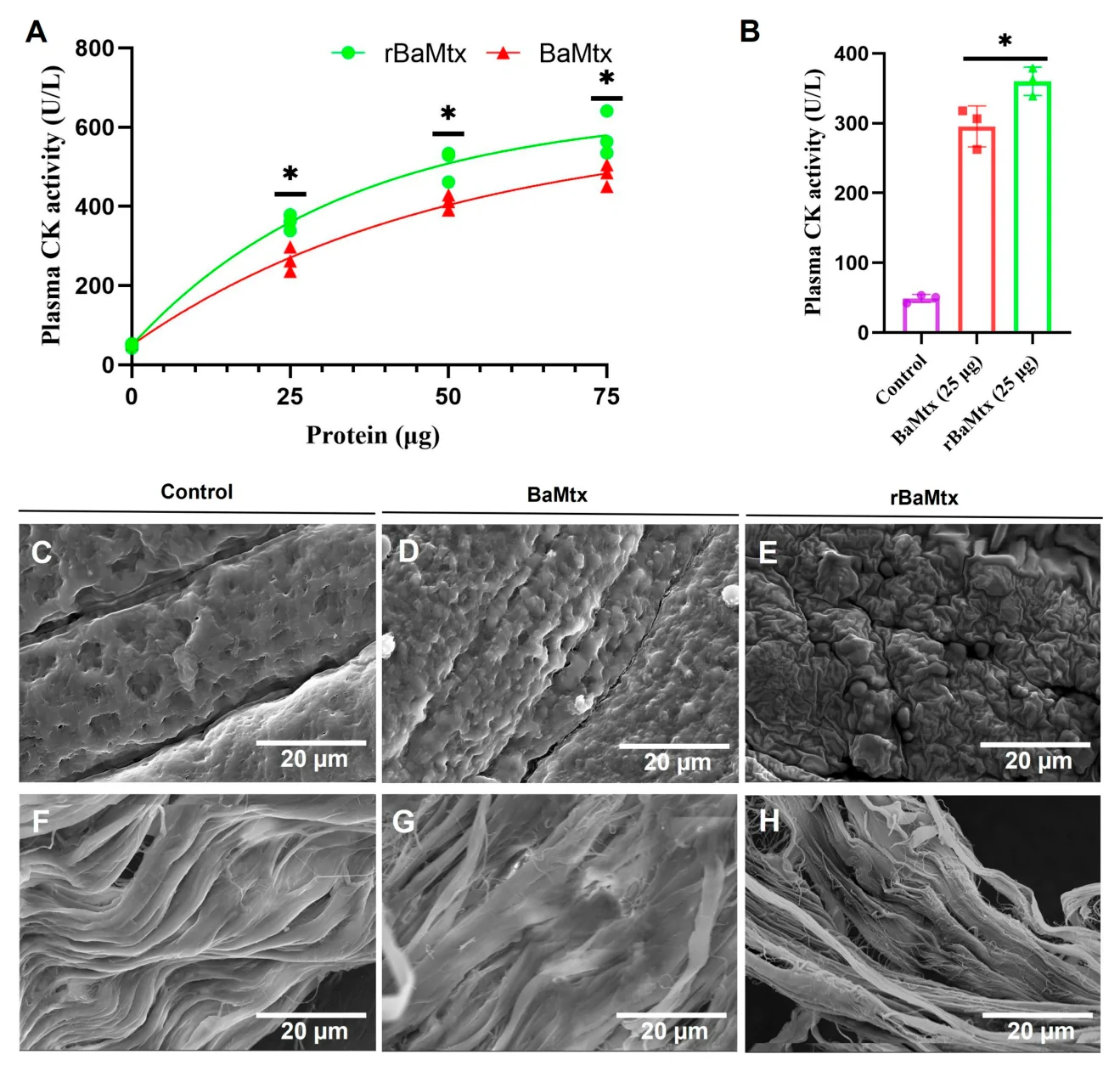
In vivo myotoxic activity and ultrastructural skeletal muscle damage induced by native and recombinant BaMtx. (**A**) Dose-dependent elevation of plasma CK activity following intramuscular injection of varying doses of native BaMtx and rBaMtx. (**B**) Comparative plasma CK activity induced by a fixed 25 µg dose, highlighting the differential myotoxicity between the native and recombinant toxins. For (**A**) and (**B**), data are expressed as mean ± SD (*n* =3). Statistical significance was determined using a Two-way ANOVA followed by test post hoc Tukey’s multiple comparisons test for (**A**), and a unpaired Student’s *t*-test for (**B**). * *p* < 0.01 indicates significant differences between the native and recombinant toxins at the specified doses. (**C**–**H**) SEM analysis of skeletal muscle tissue 1 h post-injection. Top panels (**C**–**E**) illustrate the superficial sarcolemmal topography, while bottom panels (**F**–**H**) display cross-sections of deep skeletal muscle fibers. Scale bars: 20 µm.

Intramuscular administration of native BaMtx and rBaMtx induced a dose-dependent increase in plasma CK activity across all evaluated concentrations (25, 50, and 75 µg) (Figure 2A). The recombinant toxin generated significantly higher CK levels than the native variant at every tested point, a differential myotoxic profile detailed explicitly at the 25 µg fixed dose (Figure 2B). The Minimum Myotoxic Dose (MMD) for rBaMtx was established at 9.90 µg (95% CI: 7.39–12.80), while the MMD for the native toxin was calculated at 15.53 µg (95% CI: 12.90–18.49) MMD of the native toxin.

Scanning electron microscopy performed 1 h after toxin injection revealed marked structural alterations in the skeletal muscle. Control tissues displayed parallel myofibers surrounded by a continuous sarcolemma (Figure 2C). In contrast, muscles treated with native BaMtx (Figure 2D) or rBaMtx (Figure 2E) exhibited sarcolemmal disorganization, membrane irregularities, and focal surface protrusions. Cross-sectional images further showed that control muscles preserved their characteristic parallel arrangement (Figure 2F), whereas toxin-treated tissues exhibited fiber swelling, fragmentation, and severe disruption of the normal muscular organization (Figure 2G–H).

### 2.3. In Vitro Cytotoxicity on Murine 4T1 Breast Carcinoma Cells

**Figure 3:**
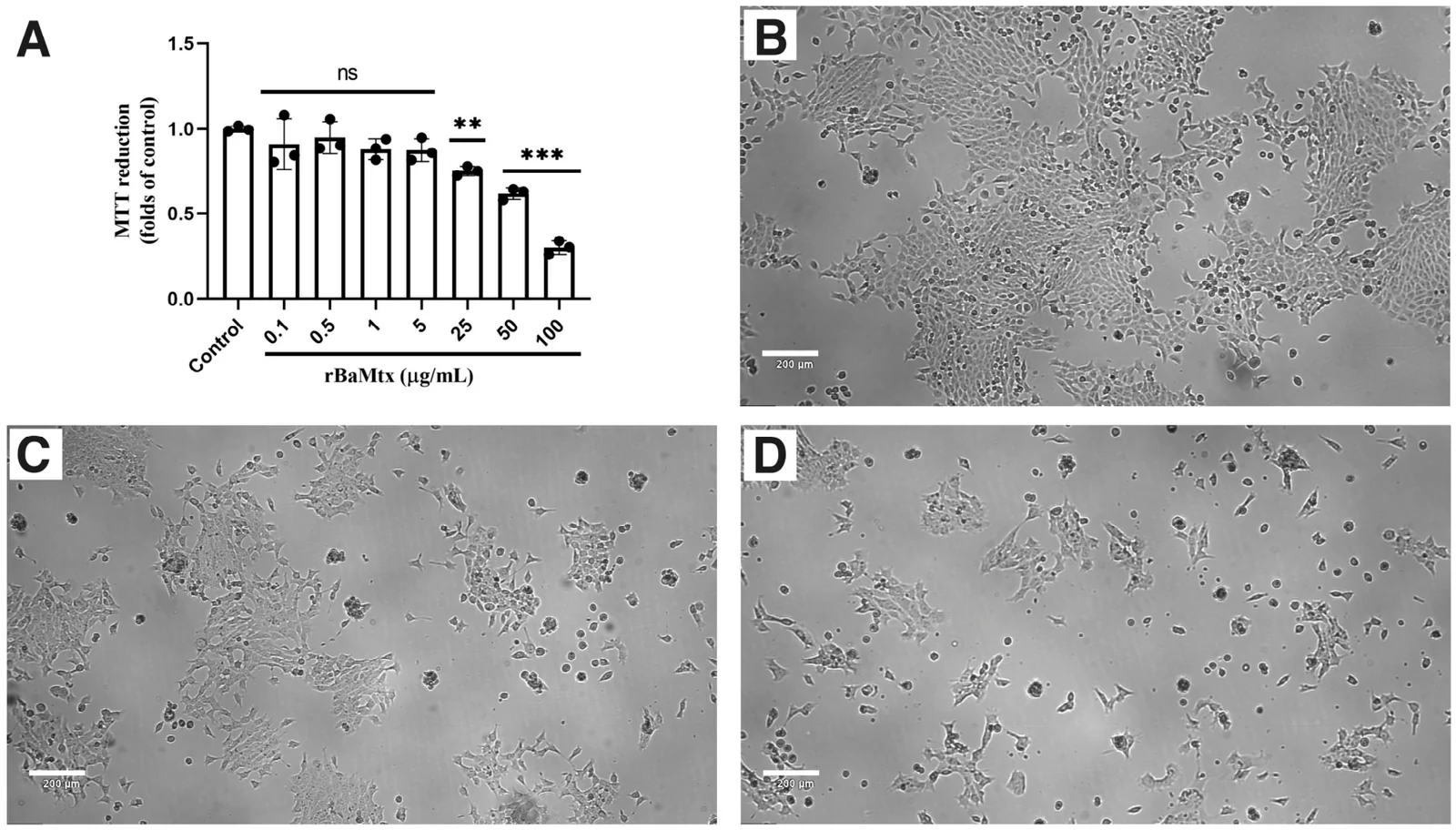
Effects of rBaMtx on metabolic activity and morphology of 4T1 murine breast carcinoma cells. (**A**) Cell viability determined by the MTT assay after 48 h of treatment with increasing concentrations of rBaMtx. Data are expressed as mean ± SD of three independent experiments (*n* = 3) and normalized to the untreated control. Statistical significance was determined using a One-way ANOVA followed by Tukey’s post hoc test (** *p* < 0.01, *** *p* < 0.001 vs. control; ns, not significant). (**B**–**D**) Representative micrographs showing dose-dependent morphological alterations in untreated cells (**B**) and cells treated with 50 (**C**) or 100 µg/mL (**D**) rBaMtx. Scale bars: 200 µm.

Exposure of 4T1 murine breast carcinoma cells to rBaMtx for 48 h resulted in a concentration-dependent reduction in metabolic activity, with statistically significant effects observed from 25 µg/mL onward (Figure 3A). Non-linear regression analysis yielded an IC50 value of 60.94 ± 2.15 µg/mL. The digital cell imaging system supported these findings, revealing progressive cell rounding, a gradual reduction in cell size, loss of confluence, reduced adhesion to the substrate, and a decrease in cell density following exposure to the toxin. Untreated cells formed a dense and highly confluent monolayer (Figure 3B), whereas exposure to 50 µg/mL induced partial loss of confluence and cell rounding (Figure 3C). At 100 µg/mL, cultures exhibited extensive cell detachment and marked disruption of their normal adherent morphology (Figure 3D).

Analysis of culture supernatants after 48 h of treatment revealed no detectable secretion of TNF-α, IL-6, or IL-10 (Table S1). In contrast, exposure to 100 µg/mL of rBaMtx induced the release of IL-1β near the assay’s detection limit (15.6–1000 pg/mL), reaching a value of 18.6 ± 1.9 pg/mL. No IL-1β was detected in the control group. Taken together, these findings indicate that metabolic and morphological effects are induced by rBaMtx even though the production of pro- and anti-inflammatory cytokines is not detected.

### 2.4. Cytotoxic Profiling in Human In Vitro Models

#### 2.4.1. Metabolic Viability Profiles

**Figure 4:**
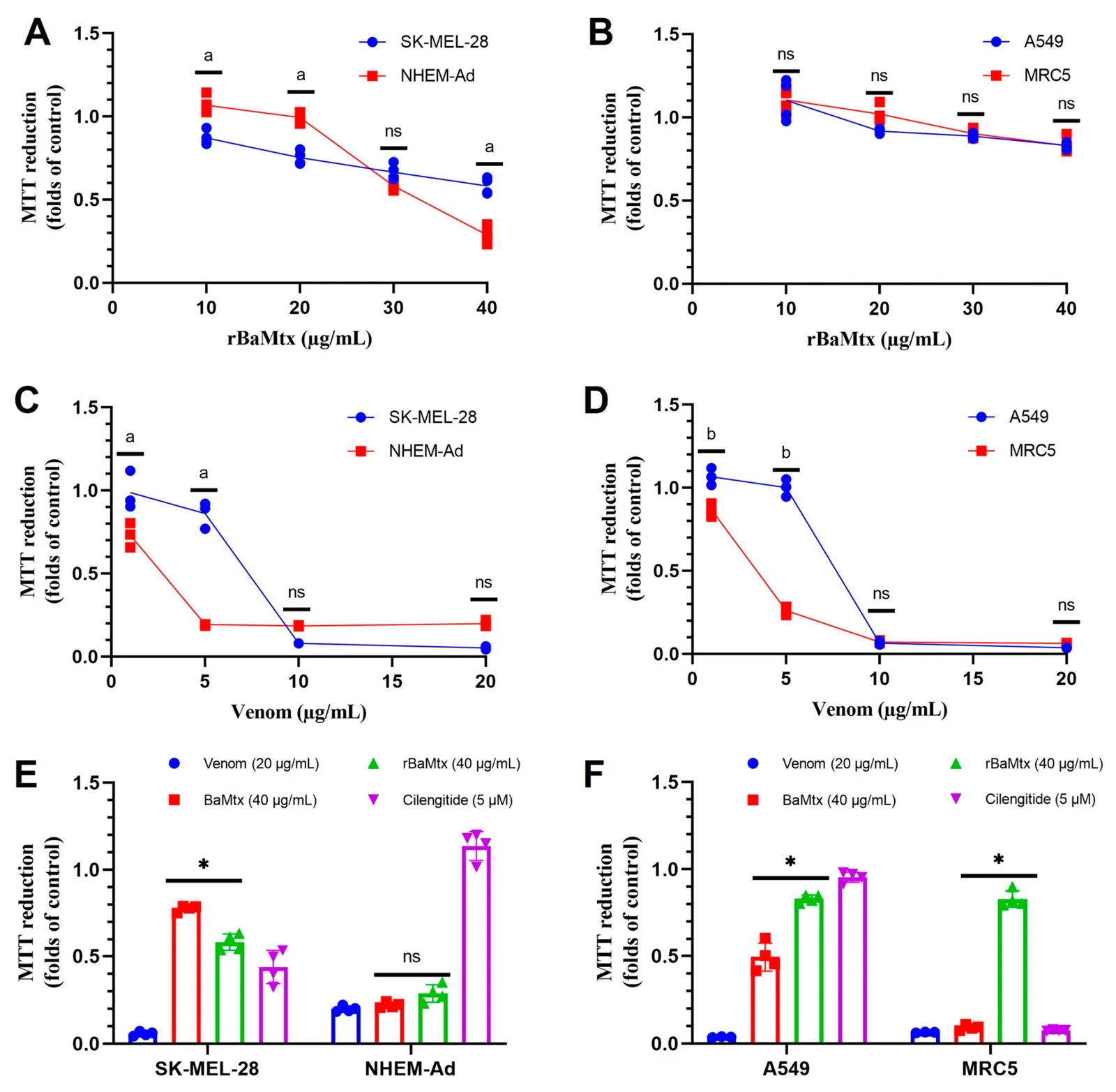
Differential effects of rBaMtx on the metabolic activity of human tumor and non-tumorigenic cell lines. Metabolic viability was assessed by the MTT assay and normalized to the vehicle control. (**A**,**B**) Dose–response curves for rBaMtx (10–40 µg/mL). (**C**,**D**) Dose–response curves for crude *B. atrox* venom (1–20 µg/mL). (**E**,**F**) Comparative metabolic viability following treatment with crude venom (20 µg/mL), native BaMtx (40 µg/mL), rBaMtx (40 µg/mL), or Cilengitide (5 µM) in skin (SK-MEL-28 and NHEM-Ad) and lung (A549 and MRC5) cell models. Data are expressed as mean ± SD (*n* = 3 for crude *B. atrox* venom, *n* = 4 for all other treatments). Statistical significance was determined by two-way ANOVA followed by Tukey’s multiple comparisons test; a, *p* < 0.01 (SK-MEL-28 vs. NHEM-Ad); b, *p* < 0.01 (A549 vs. MRC5); *, *p* < 0.01 (native BaMtx vs. rBaMtx); ns, not significant.

Exposure to rBaMtx produced distinct, tissue-dependent effects on cellular metabolic activity. In the melanoma model (Figure 4A), treatment with either 10 or 20 µg/mL significantly reduced the metabolic activity of SK-MEL-28 cells, whereas non-tumorigenic NHEM-Ad melanocytes remained largely unaffected. However, this differential response was lost at higher concentrations, as exposure to 40 µg/mL resulted in a pronounced reduction in viability in both cell types, with NHEM-Ad cells displaying greater sensitivity than the melanoma line. In contrast, the lung model exhibited a markedly different profile (Figure 4B). Increasing concentrations of rBaMtx induced a gradual decline in metabolic activity in both A549 carcinoma cells and MRC5 fibroblasts, with no significant differences between the two cell types throughout the evaluated concentration range.

Crude *B. atrox* venom produced a rapid and pronounced reduction in metabolic activity across all evaluated cell lines (Figure 4C–D). Notably, the non-tumorigenic NHEM-Ad and MRC5 cells displayed greater sensitivity than their respective tumor-derived counterparts at the lowest venom concentrations tested.

Previous literature data demonstrated, in dose–response assays, that a concentration of 40 µg/mL of native BaMtx induced cell cycle arrest and pronounced cytotoxic effects in MDA-MB-231 and MCF-7 breast cancer cell lines [8]. Based on these findings, a single concentration of 40 µg/mL of native BaMtx was selected in this study to directly compare its functional effects with rBaMtx at the same concentration (Figure 4E,F). Direct comparison of purified toxin variants at 40 µg/mL revealed important functional differences between native and recombinant BaMtx. In the melanoma model (Figure 4E), rBaMtx reduced SK-MEL-28 metabolic activity significantly more than native BaMtx, whereas both proteins strongly affected NHEM-Ad cells. Conversely, in the lung model (Figure 4F), native BaMtx induced a marked reduction in metabolic activity in both A549 and MRC5 cells, while rBaMtx exhibited a substantially attenuated effect, maintaining approximately 80% viability in both cell lines.

#### 2.4.2. Membrane Integrity and Lytic Cell Death

**Figure 5:**
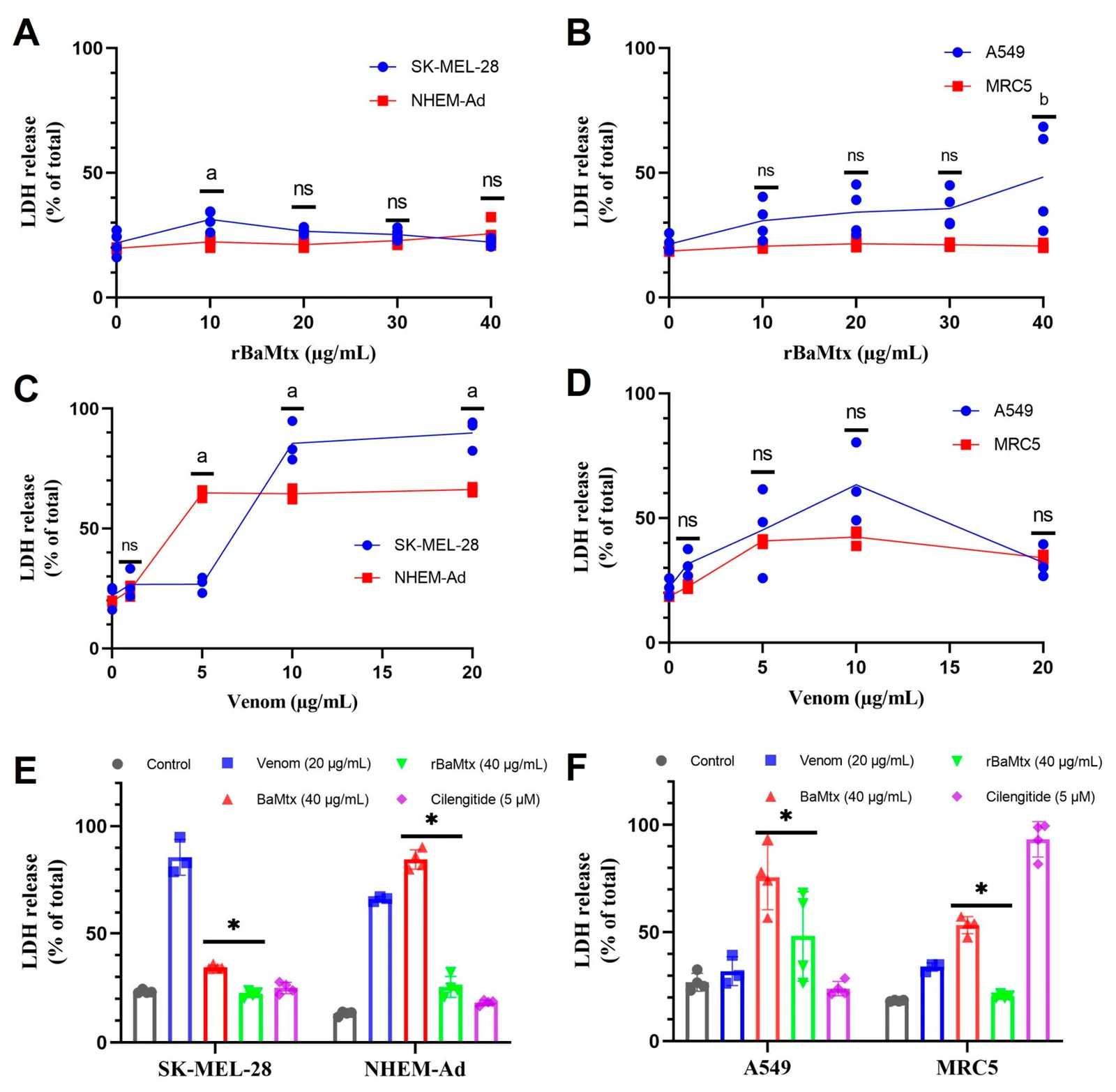
Effect of rBaMtx on membrane integrity and lytic cell death of human tumor and non-tumorigenic cell lines. Cytotoxicity was assessed by LDH release and expressed as the percentage of total LDH release. (**A**,**B**) Dose–response curves for rBaMtx (10–40 µg/mL). (**C**,**D**) Dose–response curves for crude *B. atrox* venom (1–20 µg/mL). (**E**,**F**) Comparative cytotoxicity following treatment with crude venom (20 µg/mL), native BaMtx (40 µg/mL), rBaMtx (40 µg/mL), or Cilengitide (5 µM) in skin (SK-MEL-28 and NHEM-Ad) and lung (A549 and MRC5) cell models. Data are expressed as mean ± SD (*n* = 3 for crude *B. atrox* venom, *n* = 4 for all other treatments). Statistical significance was determined using Two-way ANOVA followed by Tukey’s multiple comparisons test; a, *p* < 0.01 (SK-MEL-28 vs. NHEM-Ad); b, *p* < 0.01 (A549 vs. MRC5); *, *p* < 0.01 (native BaMtx vs. rBaMtx); ns, not significant.

Parallel assessments of membrane integrity and lytic cell death were performed by measuring LDH release into the culture medium. rBaMtx (10–40 µg/mL) demonstrated a selective non-lytic effect depending on cell type. The recombinant toxin maintained the LDH release near baseline levels (~20–30%) for both skin lineages up to the maximum dose (Figure 5A). Similarly, rBaMtx preserved membrane integrity at lower concentrations in the lung model (Figure 5B), although the 40 µg/mL dose triggered a significant elevation in LDH release specifically in A549 carcinoma cells compared to unaffected MRC5 fibroblasts, indicating a cell-line specific lytic effects.

In contrast, exposure to crude *B. atrox* venom (1–20 µg/mL) induced a robust, dose-dependent rupture of the plasma membrane. In the skin model (Figure 5C), normal NHEM-Ad melanocytes exhibited a rapid lytic response at 5 µg/mL, whereas SK-MEL-28 melanoma cells required higher concentrations (10 and 20 µg/mL) to reach maximum LDH release. In the lung model (Figure 5D), the crude venom caused comparable membrane damage in both A549 and MRC5 lines.

Further comparative analysis at fixed concentrations (40 µg/mL) explicitly defined the divergent lytic capabilities of the native and recombinant variants. In the skin model (Figure 5E), native BaMtx caused extensive membrane damage in normal NHEM-Ad cells (>80% LDH release) and moderate lysis in SK-MEL-28 cells. The recombinant toxin, however, kept LDH release at baseline levels for both lines, yielding a highly significant statistical divergence between the two preparations. This differential behavior was mirrored in the lung model (Figure 5F); while native BaMtx induced severe lysis in both A549 and MRC5 cells, rBaMtx provoked significantly less membrane damage across both tissues.

#### 2.4.3. Multiparametric Cell Density and Morphological Analysis

**Figure 6:**
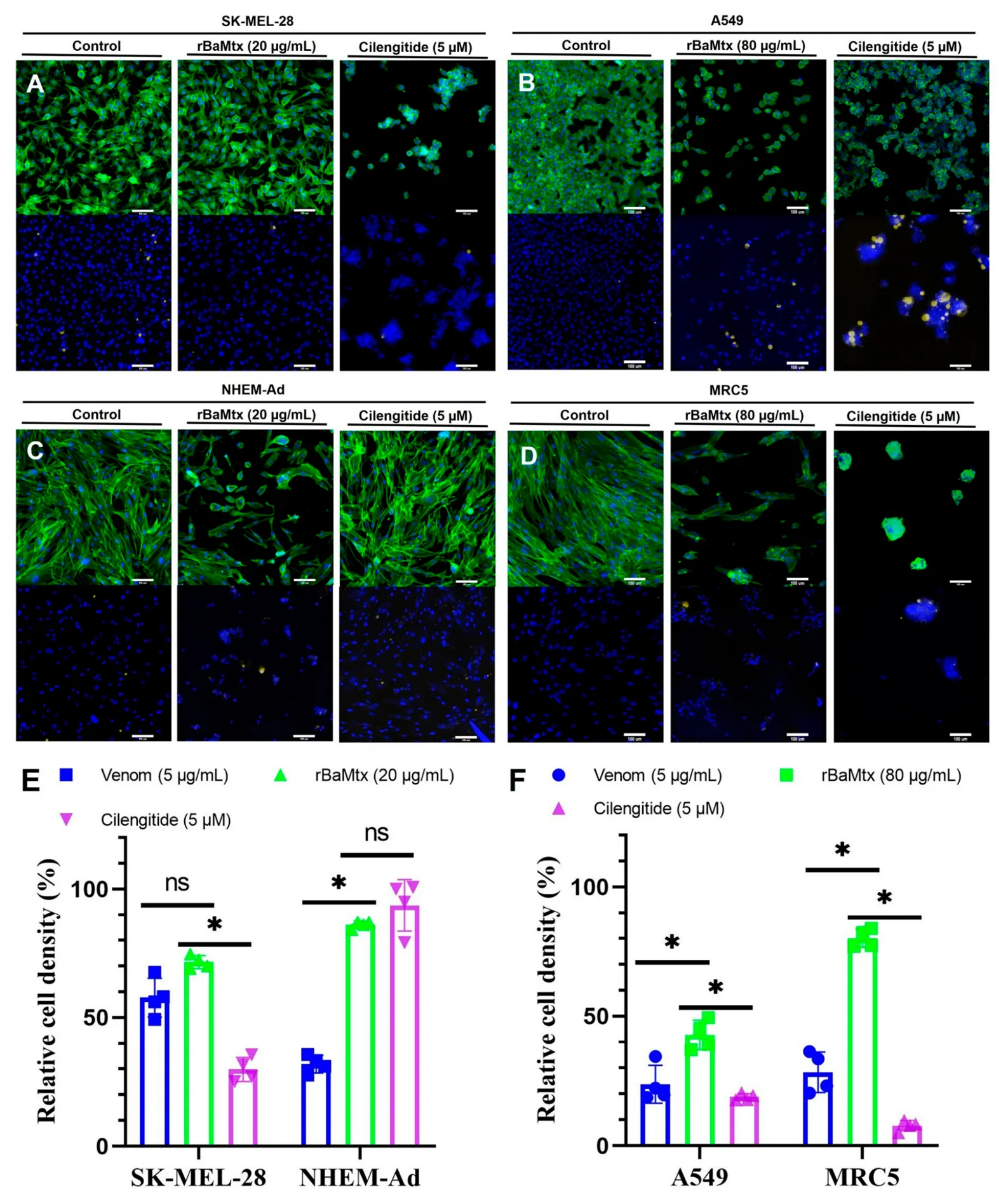
Effects of rBaMtx on morphological alterations and cell density. (**A**–**D**) Representative fluorescence images of the skin (**A**,**C**) and lung (**B**,**D**) models. Merged images show F-actin (Alexa Fluor™ 488 Phalloidin, green), nuclei (Hoechst 33342, blue), and non-viable cells (Propidium Iodide, yellow); the bottom row shows the Hoechst/PI channels only. Scale bars: 100 µm. (E, F) Relative cell density quantified via automated nuclear counting and normalized to the vehicle-treated control (100%) for the quantitative assays. (**E**) Skin model (SK-MEL-28 vs. NHEM-Ad) exposed to crude venom (5 µg/mL), rBaMtx (20 µg/mL), and Cilengitide (5 µM). (**F**) Lung model (A549 vs. MRC5) exposed to crude venom (5 µg/mL), rBaMtx (80 µg/mL), and Cilengitide (5 µM). Data are expressed as mean ± SD (*n* = 4). Statistical significance was determined using Two-way ANOVA followed by Tukey’s multiple comparisons test; * (*p* < 0.01) indicates significant differences between the specifically bracketed treatments; ‘ns’ indicates no significant difference.

The metabolic and membrane integrity profiles were further evaluated using high-content screening (HCS) (Figure 6A). Automated nuclear counting revealed distinct, tissue-specific responses to rBaMtx. Although cell counting indicated a ~25% reduction in SK-MEL-28 cells following exposure to 20 µg/mL of rBaMtx (Figure 6E), no changes in confluence were observed upon analyzing F-actin morphology (Figure 6A). Whereas the non-tumorigenic NHEM-Ad population showed no change in cell counts upon treatment (Figure 6C), it exhibited distinct morphological changes, including cell aggregation in the absence of PI nuclear staining (yellow fluorescence). On the other hand, the integrin inhibitor cilengitide reduced SK-MEL-28 cell density, accompanied by detachment, without inducing PI positivity, while having no effect on NHEM cells. In the lung model (Figure 6B), rBaMtx at 80 µg/mL severely reduced A549 carcinoma cell density with PI internalization, while preserving a significantly higher proportion of normal MRC5 fibroblasts (~85%) without widespread PI staining; however, these cells were highly aggregated (Figure 6D), suggesting cell loss without acute membrane breakdown. Similarly, cilengitide induced a decrease in cell density and cell detachment in both lung cell lines, with A549 cells exhibiting a cytolytic effect demonstrated by PI internalization (Figure 6B,D). In contrast, crude *B. atrox* venom (5 µg/mL) produced a profound reduction in cell density across all evaluated lineages (Figure 6E,F).

## 3. Discussion

Expression in *P. pastoris* enabled the successful secretion of rBaMtx, yielding a recombinant protein that retained the key biochemical and biological properties of its native counterpart [8]. The recombinant toxin displayed the expected molecular mass, lacked detectable phospholipase activity, retained strong immunoreactivity toward anti-BaMtx polyclonal antibodies, and conserved its characteristic in vivo myotoxicity, indicating that the structural determinants required for its biological activity remained largely intact after heterologous expression. This preservation may partly reflect the tag-free expression strategy adopted in this study. Although affinity tags facilitate purification and detection, they may interfere with terminal regions that are essential for the activity of Lys49-PLA_2_ homologues [7]. In these toxins, the N-terminal region is required for structural stability and proper folding, whereas the highly basic C-terminal region contains the membrane docking (MDoS) and membrane disruption (MDiS) sites responsible for membrane recognition and destabilization [18].

The successful recovery of a biologically active rBaMtx further highlights the suitability of *P. pastoris* for producing structurally complex venom proteins. The loss of antibody recognition under reducing conditions suggests that disulfide bond reduction partially disrupted conformational epitopes [19]. Importantly, rBaMtx exhibited the classic propensity of Lys49-PLA_2_s to form stable homodimers. While MALDI-TOF analysis confirmed the exact monomeric mass of 13,821 Da, non-reducing SDS-PAGE and Western blot analyses revealed a predominant dimeric assembly at ~23 kDa, which was completely dissociated into monomers under reducing conditions. This dimerization is a critical structural hallmark of native Lys49-PLA_2_s [8]. Although the correct pairing of disulfide bonds was not directly verified (e.g., by peptide mapping or crystallography), the observed molecular mass, dimerization pattern, and myotoxic activity are consistent with native-like folding; future studies could confirm disulfide connectivity using non-reduced tryptic mapping coupled to mass spectrometry. In contrast to prokaryotic hosts such as *E. coli*, where recombinant snake venom PLA_2_s often require recovery from inclusion bodies followed by in vitro refolding [12], the eukaryotic secretory pathway of *P. pastoris* promotes oxidative folding and correct disulfide bond formation in cysteine-rich proteins [20], and the successful assembly of native-like quaternary structures. Together with the tag-free strategy, these features enabled the production of a structurally and functionally competent Lys49-PLA_2_ homologue that provides a homogeneous and reproducible model for investigating the biological properties of this toxin family independently of enzymatic phospholipase activity.

Despite the complete absence of detectable phospholipase activity, rBaMtx retained in vivo myotoxicity, inducing a dose-dependent increase in plasma creatine kinase levels comparable to, and consistently higher than, those produced by native BaMtx. Because the myotoxic activity of Lys49-PLA_2_ homologues depends on the preservation of their native conformation rather than catalytic phospholipase activity [21,22], these findings indicate that rBaMtx conserved the structural determinants required for this characteristic biological effect. The slightly greater potency of the recombinant toxin may reflect the greater homogeneity of the recombinant preparation relative to native purified fractions, which naturally consist of a phenotypic pool of related microvariants.

Proteomic studies indicate that Peruvian *B. atrox* venom is highly complex, with PLA_2_s comprising 11–14% of the venom proteome [23,24]. Interestingly, while *B. atrox* venom contains multiple catalytically active Asp49-PLA_2_ isoforms, including FA3/BATXPLA006 and FA4/BATXPLA001 [25], and other Lys49-PLA_2_ gene product, FA1/BATXPLA002, has been molecularly characterized to date. However, several additional Lys49 and Asp49-PLA_2_s have been isolated from different *B. atrox* populations [26], although most lack complete primary-sequence characterization, precluding determination of whether they represent distinct Lys49 genes or allelic/post-translational variants of FA1/BATXPLA002 and BaMtx. Moreover, the notably higher in vitro potency typically observed for the crude venom occurs because the activity of the Lys49 isoform is amplified by cooperative interactions with Asp49-PLA_2_s [27] or metalloproteinases [28]. Therefore, the biological activity observed for rBaMtx, despite its greater molecular homogeneity, confirms that the recombinant protein retains the characteristic functional properties of native BaMtx.

As an initial in vitro evaluation using the murine 4T1 breast carcinoma cell line, rBaMtx produced a concentration-dependent reduction in metabolic viability, with an IC_50_ of approximately 60.94 µg/mL. This value falls within the range reported for other basic Lys49-PLA_2_ homologues, including BthTX-I and BnSP-6 [29,30]. Cells treated with 50 and 100 µg/mL rBaMtx exhibited cell shrinkage, rounding, clustering, and increased intercellular spaces. Similar morphological changes have been reported following exposure to *B. atrox* venom components and are consistent with early alterations in cellular architecture preceding extensive membrane disruption [8,31]. Collectively, these findings suggest that reduced metabolic viability is accompanied by early alterations in cellular architecture.

Exposure of 4T1 cells to the highest concentration of rBaMtx induced IL-1β release, whereas TNF-α, IL-6, and IL-10 remained undetectable. This selective cytokine profile is consistent with inflammatory responses previously reported for Lys49-PLA_2_ homologues and other snake venom toxins [32,33,34]. However, because canonical markers of inflammasome activation and pyroptosis were not evaluated, IL-1β release should be interpreted as evidence of an inflammatory response rather than a specific cell-death pathway. Extending this characterization to paired human tumor and non-tumor cell models, the combined analysis of metabolic activity (MTT), membrane integrity (LDH), and high-content imaging showed that reduced metabolic viability was not consistently accompanied by plasma membrane disruption, suggesting that the cellular effects of rBaMtx cannot be explained solely by overt membrane damage.

In the skin model, rBaMtx preferentially affected SK-MEL-28 melanoma cells at lower concentrations, reducing both metabolic activity and cell density while largely preserving the viability of non-tumorigenic NHEM-Ad melanocytes. By contrast, the lung model exhibited a different response, with A549 carcinoma cells and MRC5 fibroblasts showing comparable reductions in MTT activity despite minimal LDH release across the evaluated concentration range. These findings suggest that differences among cell models influence the response to rBaMtx.

A notable finding was the dissociation between metabolic impairment, membrane integrity, and cell density. Although high-content screening detected marked reductions in cell number, LDH release and propidium iodide incorporation remained low under most experimental conditions, indicating that extensive plasma membrane disruption was not the predominant cellular outcome. Because high-content imaging requires washing steps, reductions in cell density should be interpreted together with complementary biochemical assays rather than as direct evidence of cell death. Collectively, the MTT, LDH, propidium iodide, and imaging data reveal cell-type-selective responses, ranging from localized lytic effects to a predominantly non-lytic profile characterized by early alterations in morphology and adhesion. Similar changes in cytoskeletal organization and tissue architecture have been reported following exposure to *B. atrox* venom components [31], consistent with, though not directly demonstrating, structural remodeling as an early cellular response to venom-derived toxins. Although native BaMtx induces G1-phase arrest and apoptosis in breast cancer cells [8], these mechanisms cannot be directly extrapolated to the melanoma and lung carcinoma models examined here, emphasizing the need for future studies addressing apoptosis- and adhesion-related signaling pathways.

The differential susceptibility observed among the evaluated cell lines likely reflects differences in membrane composition and the availability of cell-surface interaction partners [35]. Lys49-PLA_2_ homologues have been proposed to interact with integrins and surface nucleolin, thereby influencing cell adhesion, migration, and internalization [36,37]. However, this study relied exclusively on metabolic (MTT), membrane integrity (LDH), and imaging-based readouts; no markers of apoptosis, mitochondrial function, or integrin/nucleolin engagement were assessed. Accordingly, these and other mechanisms discussed here remain plausible hypotheses by analogy with other Lys49-PLA2 homologues, rather than findings directly demonstrated in this work.

The differential responses observed in the present study also appear to operate within a relatively narrow concentration range. In the skin model, the preferential effect of rBaMtx on SK-MEL-28 cells was lost at the highest concentration evaluated (40 µg/mL), where non-tumorigenic melanocytes also exhibited a marked reduction in metabolic viability. This progressive loss of selectivity suggests that the cellular response to rBaMtx depends on multiple factors, including membrane composition and the availability of surface interaction partners [35,36,37,38,39,40]. At higher concentrations, the highly cationic nature of Lys49-PLA_2_ homologues may increasingly favor nonspecific interactions with the plasma membrane, thereby reducing the preferential responses observed at lower doses.

To our knowledge, this study represents the first functional evaluation of a recombinant Lys49-PLA_2_ produced in *P. pastoris* in the context of venomics-based cancer drug discovery. Compared with native BaMtx and crude *B. atrox* venom, rBaMtx elicited cell-type selective effect while preserving characteristic biological activity, a difference that may reflect the greater structural homogeneity of the recombinant protein relative to native venom, to native purified fractions, which may contain allelic or post-translationally modified microvariants of the single Lys49-PLA_2_ gene product described in this venom (see discussion above) [25]. It should be noted that crude venom was evaluated at lower concentrations (1–20 µg/mL) than rBaMtx and native BaMtx (10–40 µg/mL), reflecting the higher potency of the venom mixture due to synergistic interactions among multiple toxin families [27,28]. This activity could not be normalized to a BaMtx-equivalent dose without direct quantification of the Lys49-PLA_2_ component within the venom pool; therefore, crude venom comparisons should be interpreted as mixture-level phenotypes rather than isolated Lys49-PLA2 activity. In contrast, native BaMtx and rBaMtx were directly compared at the same concentration (40 µg/mL; Figure 4E,F and Figure 5E,F), enabling a dose-controlled evaluation of differences between protein preparations. The inclusion of paired tumor and non-tumor cell models further demonstrated that the biological effects of Lys49-PLA_2_ homologues are strongly influenced by tissue context, underscoring the importance of evaluating venom-derived molecules across multiple biological settings to better define their selectivity and therapeutic potential [41]. These findings provide a framework for future studies aimed at identifying the molecular determinants underlying the context-dependent activities of this toxin family.

## 4. Conclusions

This study demonstrates that *P. pastoris* KM71 enables the production of a recombinant BaMtx that preserves the key biochemical, antigenic, and biological properties of its native counterpart. Despite lacking detectable phospholipase activity, rBaMtx retained pronounced myotoxicity and elicited context-dependent responses across tumor and non-tumor cell models characterized by early alterations in cell morphology, adhesion, and metabolic activity rather than widespread membrane disruption. These findings support the concept that catalytically inactive Lys49-PLA_2_ homologues modulate cellular behavior through mechanisms extending beyond direct membrane lysis. Overall, recombinant rBaMtx provides a reproducible and scalable model for investigating the intrinsic biological properties of Lys49-PLA_2_ homologues independently of the complexity of native venom preparations, further establishing *P. pastoris* as a robust platform for future mechanistic and biomedical studies of this toxin family.

## 5. Materials and Methods

### 5.1. Venom Collection and Experimental Animals

Crude venom was pooled from three adult *Bothrops atrox* specimens (two males and one female) originating from Pucallpa, Peru, and maintained in captivity for 2–3 years at the “Oswaldo Meneses” Serpentarium of the Natural History Museum, Universidad Nacional Mayor de San Marcos (UNMSM, Lima, Peru). Venom was obtained by manual extraction, lyophilized, and stored at −20 °C until use. Freshly collected venom was preserved in RNAlater™ (Invitrogen, USA) and used as a source of extracellular RNA for cDNA synthesis, following the strategy previously validated for Peruvian *Bothrops* venoms. A portion of the lyophilized venom pool was used for purification of native BaMtx, which served as the reference protein throughout this study.

Male BALB/c mice (18–20 g) were obtained from the Instituto Nacional de Salud (INS, Lima, Peru) and maintained under standard laboratory conditions with free access to food and water. Animals were used exclusively for the evaluation of myotoxic activity. All procedures involving venomous snakes and experimental animals were approved by the Bioethics Committee of the Facultad de Ciencias Biológicas-UNMSM (Act No. 239-2026-CBE-FCB-UNMSM).

### 5.2. Protein and Nucleic Acid Quantification

Protein concentrations in crude *B. atrox* venom and extracellular fractions from *P. pastoris* culture supernatants were determined by the Lowry method [42]. Purified BaMtx and rBaMtx were quantified using a Qubit™ Fluorometer (Invitrogen™, Carlsbad, CA, USA) with the Qubit™ Protein Assay Kit (Cat# Q33212). RNA and DNA concentrations were measured using the Qubit™ RNA HS (Cat# Q32852) and dsDNA HS (Cat# Q32854) Assay Kits, respectively. Nucleic acid purity was assessed by A260/A280 absorbance using a NanoDrop™ 2000 spectrophotometer (Thermo Fisher Scientific, Waltham, MA, USA).

### 5.3. Recombinant Construct Design

Extracellular RNA was isolated directly from freshly collected *B. atrox* venom preserved in RNAlater™, following a strategy previously validated for Peruvian *Bothrops* venoms [43]. First-strand cDNA synthesis was performed using the SuperScript™ First-Strand Synthesis System (Invitrogen™, Cat# 18091050). The coding sequence corresponding to mature BaMtx was amplified by PCR using Platinum™ SuperFi II Green PCR Master Mix (Invitrogen™, Cat# 12368010) and primers incorporating SnaBI and AvrII restriction sites: 5′-GCCTACGTAACCTGTGGCAATTGGGGAAG-3′ (forward) and 5′-ACTCCTAGGTTAGCATGGCTCTGCCTTCT-3′ (reverse).

The purified PCR product and the *Pichia pastoris* expression vector pPIC9 were digested with SnaBI and AvrII and ligated using Anza™ T4 DNA Ligase according to the manufacturer’s instructions. The resulting construct, designated pPIC9-rBaMtx, was transformed into chemically competent Escherichia coli TOP10F′ cells by heat shock. Recombinant clones were selected in LB agar supplemented with ampicillin (50 µg/mL), propagated overnight at 37 °C with agitation (200 rpm), and plasmid DNA was purified using the PureLink™ Quick Plasmid Miniprep Kit (Invitrogen™, Cat# K210011). The correct in-frame insertion and sequence integrity of the BaMtx cDNA were confirmed by Oxford Nanopore sequencing on a MinION Mk1C platform (Oxford Nanopore Technologies, Oxford, UK) with an R9.4.1 flow cell and SQK-RBK004 kit. Flye assembly (v2.9.6) and >40× coverage confirmed 100% sequence identity.

### 5.4. Heterologous Expression

The recombinant expression vector was linearized with the restriction enzyme SacI (Invitrogen™ #IVGN0206) and transformed into *Pichia pastoris* strain KM71 by electroporation, according to the manufacturer’s protocol (Invitrogen™ #K1740-01). Transformants were selected on minimal dextrose (MD) agar plates based on their His+ phenotype. Selected colonies were screened by direct colony PCR using the Platinum™ SuperFi II Green PCR Master Mix (Invitrogen™ #12369010) with universal AOX1 primers to confirm the genomic integration of the rBaMtx expression cassette.

Positive clones were initially cultured in 100 mL of BMGY medium at 30 °C and 300 rpm in 1 L baffled flasks until the culture reached an OD600 of 2–6. Cells were harvested by centrifugation at 3000 × g for 5 min and subsequently resuspended in 100 mL of BMMY medium to induce recombinant toxin expression under the control of the AOX1 promoter.

The induction phase was maintained at 30 °C and 300 rpm for 7 days. Cultures were supplemented daily with methanol to a final concentration of 0.5% (*v*/*v*) to sustain continuous expression. Daily culture aliquots were collected to monitor the rBaMtx secretion profile. Cell-free supernatants were precipitated using the methanol–chloroform protein precipitation method [44] and analyzed by 15% SDS-PAGE [45]. At the end of the induction period, the bulk culture was centrifuged at 3000× *g* for 10 min at 4 °C to pellet the yeast cells. The clarified supernatant containing secreted rBaMtx was carefully collected, lyophilized, and stored at 4 °C until purification.

### 5.5. Purification of Native BaMtx and rBaMtx

Native BaMtx was purified from approximately 100 mg of pooled lyophilized *B. atrox* venom as previously described [8] and used as the reference protein throughout this study.

For recombinant toxin purification, the lyophilized *P. pastoris* secretome (200 mg; total yield: ~12.5 g from 200 mL of induced culture) was dissolved in 2 mL of 50 mM Tris-HCl buffer (pH 7.0) to isolate the recombinant toxin (rBaMtx). The suspension was centrifuged at 12,000× *g* for 20 min at 4 °C to remove insoluble aggregates, and the resulting supernatant was filtered through a 0.22 µm membrane. The clarified sample was applied to a CM-Sephadex C-25 cation-exchange column (1.4 × 32 cm) previously equilibrated with the same buffer. Elution was performed using a linear NaCl gradient from 0 to 1.0 M.

Eluted fractions were screened by indirect ELISA using polyclonal anti-BaMtx antibodies previously generated against the native toxin [8]. Fractions exhibiting the highest immunoreactivity were pooled, desalted, and concentrated using Amicon Ultra centrifugal filters (10 kDa molecular weight cut-off). Protein concentration was determined as described above, and the purity of rBaMtx was assessed by reverse-phase HPLC (RP-HPLC) using an Agilent 1260 Infinity II system equipped with a diode array detector (Agilent Technologies, Santa Clara, CA, USA). Samples (100 µL, 0.42 mg/mL in 0.1% TFA) were injected onto a Zorbax 300SB-C18 column (4.6 × 150 mm, 5 µm) and eluted at a flow rate of 1 mL/min with a linear gradient from 0 to 100% acetonitrile containing 0.1% TFA over 30 min. Elution profiles were continuously monitored at 215 and 280 nm.

The purified protein was resolved by SDS-PAGE and electrotransferred onto a 0.22 µm nitrocellulose membrane for immunodetection. The membrane was probed with primary anti-BaMtx antibodies (1:1000 dilution) for 1 h at room temperature. The blotted membrane was then subjected to standard washing steps and incubated with a horseradish peroxidase (HRP)-conjugated anti-rabbit IgG secondary antibody (1:5000 dilution). Immunoreactive bands were visualized using 3,3′-diaminobenzidine (DAB) as the chromogenic substrate.

### 5.6. Biochemical Characterization of rBaMtx

The intact molecular mass of rBaMtx was determined by matrix-assisted laser desorption/Ionization time-of-flight mass spectrometry (MALDI-TOF MS) using a Microflex LT/SH instrument (Bruker Daltonics, Bremen, Germany). The recombinant toxin was adjusted to 0.5 mg/mL in 0.1% TFA and mixed at a 1:1 ratio (*v*/*v*) with a matrix solution consisting of α-cyano-4-hydroxycinnamic acid dissolved in 30% acetonitrile containing 0.1% TFA. The mixture was spotted onto a target plate using the dried-droplet method. Mass spectra were acquired in positive linear mode using an acceleration voltage of 19.9 kV, a laser repetition rate of 60 Hz, and an extraction delay of 110 ns. The analysis was performed to determine the intact molecular mass of rBaMtx and compare it with the theoretical mass predicted from the BaMtx amino acid sequence.

Catalytic PLA_2_ activity of rBaMtx, BaMtx, and *B. atrox* venom was evaluated under identical conditions using the chromogenic substrate 4-nitro-3-(octanoyloxy)benzoic acid (4-NOBA) as previously described [46]. Reactions were performed in Tris-HCl buffer (10 mM, pH 8.0) containing 100 mM NaCl, 10 mM CaCl_2_, protein sample, and 3 mM 4-NOBA. Substrate hydrolysis was monitored at 37 °C for 60 min by measuring absorbance at 405 nm with background correction at 600 nm.

### 5.7. Assessment of Myotoxicity and Muscle Ultrastructural Damage

To evaluate whether rBaMtx retained a hallmark biological activity of the native toxin, acute in vivo myotoxicity was assessed and compared with native BaMtx. Groups of BALB/c mice (*n* = 3 per treatment) received intramuscular injections of BaMtx or rBaMtx (25, 50, or 75 µg in 50 µL of phosphate-buffered saline, PBS) into the right gastrocnemius muscle. Control animals received PBS alone. After 3 h, blood samples were collected from the submandibular vein into tubes containing 4% sodium citrate, and plasma was obtained by centrifugation. Creatine kinase (CK) activity was determined using a kinetic-enzymatic assay kit (Química Clínica Aplicada, Tarragona, Spain) according to the manufacturer’s instructions and expressed as U/L [47].

For ultrastructural analysis, an independent cohort of mice (*n* = 3 per group) received intramuscular injections of 25 µg of either native BaMtx or rBaMtx. Animals were euthanized 1 h after injection, and gastrocnemius muscles were excised [48]. A representative tissue sample from one mouse per treatment group underwent fixation in 2% glutaraldehyde in 0.1 M Sørensen phosphate buffer (pH 7.2) for 24 h at 4 °C. dehydrated through graded ethanol solutions and dried at the critical point using liquid CO_2_. Samples were mounted on aluminum stubs, sputter-coated with gold, and examined using an Inspect S50 scanning electron microscope (FEI Company, Hillsboro, OR, USA) operated at 12.5 kV under high vacuum to evaluate ultrastructural alterations in skeletal muscle fibers and sarcolemmal integrity.

### 5.8. Anticancer Activity Assays

#### 5.8.1. Screening in a Murine Tumor Cell Model

The effect of rBaMtx on cellular metabolic was evaluated on the 4T1 murine triple-negative breast carcinoma cell line (Cytion #300300). 4T1 cells were cultured in RPMI-1640 medium supplemented with 10% FBS. Cells were seeded in 96-well flat-bottom plates at a density of 2000 cells/well for the metabolic viability assay and incubated for 24 h. The culture medium was then replaced with fresh supplemented medium containing varying concentrations of rBaMtx (0.1, 0.5, 1, 5, 25, 50, and 100 µg/mL). Control wells received only the vehicle medium.

After 48 h of treatment at 37 °C in a humidified atmosphere with 5% CO_2_, cellular metabolic activity was determined using the MTT assay. Briefly, cells were incubated with MTT solution (0.5 mg/mL) for 4 h in the dark, the resulting formazan crystals were dissolved in DMSO (Sigma-Aldrich), and absorbance was measured at 570 nm with background correction at 630 nm using an EPOCH-2 microplate reader (BioTek Instruments Inc., USA). The monolayer was examined using a Millicell^®^ Digital Cell Imager (Millipore, USA) at 20× magnification.

To investigate whether treatment with rBaMtx induces the production of pro- and anti-inflammatory cytokines, the concentrations of IL-1β, TNF-α, IL-6, and IL-10 in the culture supernatants were quantified using commercial sandwich ELISA kits (Invitrogen™), following the manufacturer’s instructions. Flat-bottom microplates were coated overnight at 4 °C with the respective capture antibodies diluted in the coating buffer. Collected culture supernatants (100 µL) from the rBaMtx treatment (0.1, 0.5, 1, 5, 25, 50, and 100 µg/mL) were added to the wells following standard blocking and washing steps. All samples were analyzed in triplicate, and cytokine levels with absorbance values below the assay detection limit were considered not detected (ND). Cytokine concentrations were calculated using standard curves generated for each analyte. Absorbance was recorded at 450 nm with a differential filter at 570 nm using the EPOCH-2 spectrophotometer.

#### 5.8.2. Human Cell Lines

The human tumor-derived cell lines SK-MEL-28 melanoma (RRID: CVCL_0526; ATCC #HTB-72) and A549 lung carcinoma (RRID: CVCL_0023; ATCC #CCL-185) were obtained from the American Type Culture Collection (ATCC, Manassas, VA, USA). Two non-tumorigenic cell models were used as physiological controls: MRC-5 human lung fibroblasts (RRID: CVCL_0440; ATCC #CCL-171) and primary normal human epidermal melanocytes (NHEM-Ad; Lonza, Walkersville, MD, USA).

SK-MEL-28 and A549 cell lines were cultured in Dulbecco’s Modified Eagle High Glucose Medium (DMEM; Gibco, Grand Island, NY, USA) supplemented with 10% (*v*/*v*) fetal bovine serum (FBS, São Paulo, SP, Brazil) and sodium bicarbonate (Sigma-Aldrich, St. Louis, MO, USA). The primary NHEM-Ad cells were maintained in Melanocyte Basal Medium (MBM-4; Lonza) supplemented with the recommended MGM-4 BulletKit additives containing calcium chloride, phorbol myristate acetate (PMA), recombinant human basic fibroblast growth factor (rhFGF-B), bovine pituitary extract (BPE), hydrocortisone, and gentamicin/amphotericin (GA-1000) according to the manufacturer’s guidelines. Finally, MRC-5 fibroblasts were cultured with Minimum Essential Medium (MEM, Gibco, Grand Island, NY, USA) supplemented with 10% FBS. All cell lines were maintained at 37 °C in a humidified atmosphere with 5% CO2, using a CellXpert C170i incubator (Eppendorf, Hamburg, Germany).

#### 5.8.3. Cytotoxicity and Metabolic Viability Screening

SK-MEL-28, NHEM-Ad, A549, and MRC-5 cells were seeded in 96-well flat-bottom plates (Kasvi, Parana, Brazil) at densities of 3 × 103, 6 × 103, 5 × 10^3^, and 1 × 10^4^ cells/well, respectively, in a final volume of 100 µL of their corresponding complete culture medium. Plates were incubated at 37 °C in a humidified atmosphere with 5% CO_2_ for 24 h to allow cell adhesion. Subsequently, the medium was carefully removed and replaced by 100 µL of fresh medium, followed by the addition of 50 µL of the test compounds (previously diluted in fresh culture medium), reaching a final volume of 150 µL per well.

Primary treatments for the human cell models consisted of varying concentrations of rBaMtx (10, 20, 30, 40 and 80 µg/mL). Purified native BaMtx (40 µg/mL), and *B. atrox* crude venom (1, 5, 10 and 20 µg/mL) were included as comparative controls for native biological activity. The 40 µg/mL for the native toxin was selected based on previous reports demonstrating a 50% reduction in MCF7 tumor cell viability [8]). The integrin antagonist cilengitide (5 µM) was also evaluated as reference inhibitor to contrast the phenotypic and metabolic profiles.

Rigorous vehicle controls were incorporated to ensure that the observed cellular responses were not influenced by the sample solvents. These controls consisted of Tris-HCl buffer (pH 7.0), as well as 2% (*v*/*v*) DMSO, matching the maximum solvent concentrations present in the respective protein and chemical treatments.

Treated plates were incubated under standard conditions (37 °C, 5% CO_2_) for 48 h. All experimental conditions were performed in independent biological triplicates. Culture supernatants were carefully collected and transferred to new plates for the lactate dehydrogenase (LDH) release assay at the end of the treatment period, while the remaining adherent cells were utilized for the evaluation of metabolic viability via the MTT assay.

#### 5.8.4. Membrane Integrity and Lactate Dehydrogenase (LDH) Release Assay

To determine whether rBaMtx induced membrane damage, cell membrane integrity was assessed by quantifying LDH release upon cell lysis into the culture medium. It was performed using the CytoTox 96^®^ Non-Radioactive Cytotoxicity Assay (Promega, Madison, WI, USA), following the manufacturer’s instructions. Briefly, 50 µL of the previously collected culture supernatants were transferred to a new 96-well microplate, followed by the addition of 50 µL of the CytoTox 96^®^ substrate reagent to each well. The plate was protected from light and incubated for 30 min at room temperature before adding 50 µL of Stop Solution per well. Absorbance was recorded at 490 nm using a microplate reader within 1 h of stopping the reaction. The percentage of cytotoxicity was calculated relative to the maximum LDH release obtained from fully lysed with background absorbance subtracted from all values. An experimental response is considered non-lytic when LDH release shows no statistically significant difference (*p* > 0.05) compared to the vehicle control.

#### 5.8.5. Metabolic Viability Assay (MTT)

Metabolic cell viability, reflecting mitochondrial oxidoreductase activity, was quantified using the 3-(4,5-dimethylthiazol-2-yl)-2,5-diphenyltetrazolium bromide (MTT) reduction assay (Sigma-Aldrich, St. Louis, MO, USA). Following the removal of the culture supernatants for the LDH assay, 100 µL of MTT solution (0.5 mg/mL in culture medium) was added to the remaining adherent cells. The plates were protected from light and incubated for 3 h at 37 °C in a humidified 5% CO2 incubator. The unreacted MTT solution was carefully discarded, and the intracellular blue formazan crystals were solubilized by adding 100 µL of DMSO per well. Absorbance was measured at 540 nm using the microplate reader. Results were expressed as the percentage of cell viability relative to the control (untreated) group.

#### 5.8.6. High-Content Screening (HCS) for Live/Dead and Morphological Analysis

Cells were seeded in 96-well microplates from Greiner (Bio-One 655986, Advanced, Frickenhausen, Germany) for the study of cellular viability and structural alterations by High-Content Screening (HCS) technology. First, for Live/Dead assay, treated cell lines were incubated with the cell-permeable DNA-intercalating dye Hoechst 33342 (5 µM) for 1 h at 37 °C to stain all nuclei. Subsequently, propidium iodide (PI; Sigma-Aldrich) was added at a final concentration of 100 µM to specifically identify non-viable cells characterized by the loss of plasma membrane integrity. The plate was immediately subjected to automated image acquisition using the ImageXpress^®^ Micro Confocal High-Content Imaging System (Molecular Devices, San Jose, CA, USA) to quantify the live and dead cell populations. A total of 9 fields per well were acquired at 20× magnification, and this acquisition setup was standardized to minimize the exposure time of live cells to propidium iodide. Image analysis was subsequently performed using MetaXpress^®^ Acquisition & Analysis Software version 6.7.2.290 (Molecular Devices, San Jose, CA, USA).

After HCS images acquisition of the Live/Dead assay, cells were washed three times with 1X PBS to remove residual fluorophores. The cells were then fixed for subsequent cytoskeletal analysis by adding 3.5% formaldehyde (Merck, Darmstadt, Germany) in 1X PBS for 15 min at room temperature. After three 5 min washes with 1X PBS to remove the fixative, the cell membranes were permeabilized using 0.1% Triton™ X-100 (Sigma-Aldrich) in 1X PBS for 15 min at 4 °C, followed by three additional 5 min washes with 1X PBS.

Permeabilized samples were incubated with a blocking solution containing 5% bovine serum albumin (BSA; Sigma-Aldrich) to prevent non-specific fluorophore binding. The actin cytoskeleton (F-actin filaments) was then selectively stained using Alexa Fluor™ 488 Phalloidin (Invitrogen, Carlsbad, CA, USA) diluted 1:500 from the commercial stock solution. The plates were protected from light and incubated for 1 h at room temperature. Finally, the staining solutions were removed, and the cells were washed thoroughly with 1X PBS to eliminate unbound fluorophores. The samples were maintained submerged in 1X PBS for automated image acquisition and multiparametric morphological analysis using the HCS system. A total of 16 fields per well were acquired at 20× magnification. Image Analysis was subsequently performed using the MetaXpress^®^Acquisition & Analysis Software version 6.7.2.290 (Molecular Devices, San Jose, CA, USA). Fluorescence data were normalized using untreated negative controls, while unstained controls were used to normalize background fluorescence and distinguish nonspecific background signals from the specific fluorescence quantified.

### 5.9. Statistical Analysis

Quantitative data are expressed as the mean ± standard deviation (SD). Statistical differences among experimental groups were analyzed according to the experimental design. Single-variable comparisons across multiple groups were analyzed using a One-way Analysis of Variance (ANOVA) followed by Tukey’s post hoc test. When comparing the interaction of two variables simultaneously (e.g., distinct cell lineages and escalating toxin concentrations), a Two-way ANOVA was applied, followed by Tukey’s multiple comparisons test. Direct comparisons between two specific independent groups (e.g., baseline vs. single treatment) were evaluated using an unpaired Student’s *t*-test. A *p*-value < 0.01 was considered statistically significant. All statistical analyses and data visualizations were performed using GraphPad Prism software version 8.0.1 (GraphPad Software, San Diego, CA, USA).

### 5.10. Ethical Statement

All experimental protocols involving animal models and *B. atrox* venom extraction were conducted in strict accordance with national and institutional guidelines for animal care and welfare. The procedures were formally reviewed and approved by the Ethics Committee of the Faculty of Biological Sciences at the Universidad Nacional Mayor de San Marcos (UNMSM, Lima, Peru) under the official approval code N° 239-2026-CBE-FCB-UNMSM.

## Data Availability

The original contributions presented in this study are included in the article/Supplementary Material. Further inquiries can be directed to the corresponding authors.

## Supplementary Materials

The following supporting information can be downloaded at: https://www.mdpi.com/article/10.3390/toxins18080338/s1, Figure S1: Map and sequence verification of the recombinant expression vector pPIC9-rBaMtx; Figure S2: PCR screening of recombinant *Pichia pastoris* KM71 transformants; Figure S3: Chromatographic purification of native BaMtx and comparative PLA_2_ enzymatic activity. Table S1. Cytokine concentrations (pg/mL) in 4T1 cell cultures treated with rBaMtx.

## Abbreviations

The following abbreviations are used in this manuscript:

PLA_2_	Phospholipase A2
BaMtx	Lys49 PLA_2_ homologue of *Bothrops atrox* venom
rBaMtx	Recombinant BaMtx
SVMP	Snake venom metalloprotease
SVSP	Snake venom serine protease
AOX1	Alcohol oxidase 1 (promoter)
cDNA	Complementary DNA
RP-HPLC	Reverse-phase high-performance liquid chromatography
MALDI-TOF MS	Matrix-assisted laser desorption/ionization time-of-flight mass spectrometry
ELISA	Enzyme-linked immunosorbent assay
HRP	Horseradish peroxidase
MTT	3-(4,5-dimethylthiazol-2-yl)-2,5-diphenyltetrazolium bromide
CK	Creatine kinase
SEM	Scanning electron microscopy
BSA	Bovine serum albumin
