# Supplementary material for: Recombinant BaMtx Preserves the Biological Properties of a Lys49 Phospholipase A2 from Bothrops atrox and Reveals Differential Responses in Tumor and Non-Tumor Cell Models
Source: Toxins (Basel). 2026 Aug 2;18(8):338. doi: 10.3390/toxins18080338 (PMC13517361; doi:10.3390/toxins18080338)
Supplement: Supplementary file 1 [file toxins-18-00338-s001.zip › toxins-4440909-supplementary.pdf]

Supplementary Materials: Recombinant BaMtx Preserves the Biological Properties of a Lys49 Phospholipase A2 from Bothrops atrox and Reveals Differential Responses in Tumor and Non-Tumor Cell Models

Daniel Torrejón, Angie Regalado, Alex Proleón, Víctor Otárola, Fanny Lazo, Edith Rodríguez, Erasmo Colona, Libertad Alzamora, Jherson Cisneros, Jacquelyne Zarria-Romero, Myriam Paola Alvarez-Flores, Renata Nascimento, Thatiana Corrêa de Melo, Ronnie G. Gavilan, Javier Cárdenas, Félix A. Urra, Dan E. Vivas-Ruiz and Armando Yarlequé

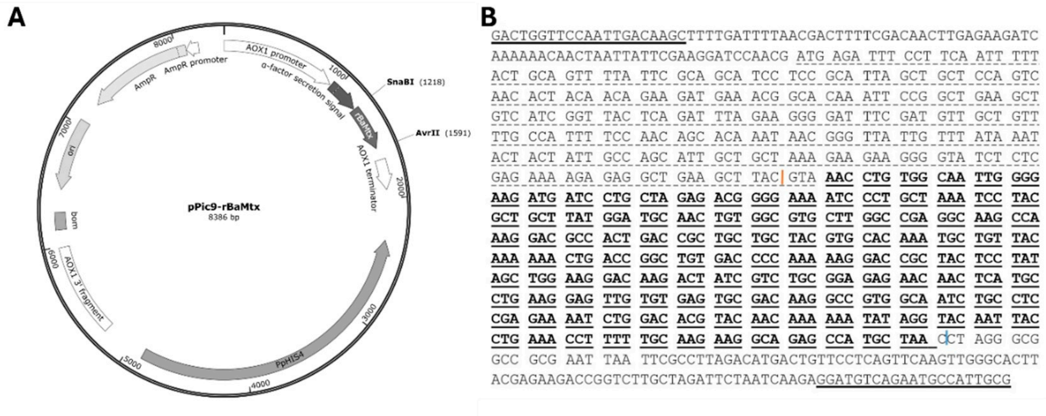

Figure S1. Map and sequence verification of the recombinant expression vector pPIC9-rBaMtx. (A) Plasmid map of the pPIC9-rBaMtx construct (8386 bp). Key genetic features are indicated, including the AOX1 promoter and terminator, the Saccharomyces cerevisiae α-factor secretion signal, the rBaMtx coding sequence, and the restriction enzyme sites (SnaBI and AvrII) utilized for cloning. (B) Nucleotide sequence of the expression cassette obtained via Oxford Nanopore sequencing using AOX specific primers. The continuous solid underlines at the 5' and 3' extremities denote the AOX forward and reverse primer binding sites, respectively. The dashed underline indicates the α-factor secretion signal sequence. The bold and underlined sequence corresponds to the inserted rBaMtx coding region, ending with the TAA stop codon. Vertical lines indicate the precise cleavage sites for the SnaBI (orange, TAC|GTA) and AvrII (light blue, C|CTAGG) restriction endonucleases.

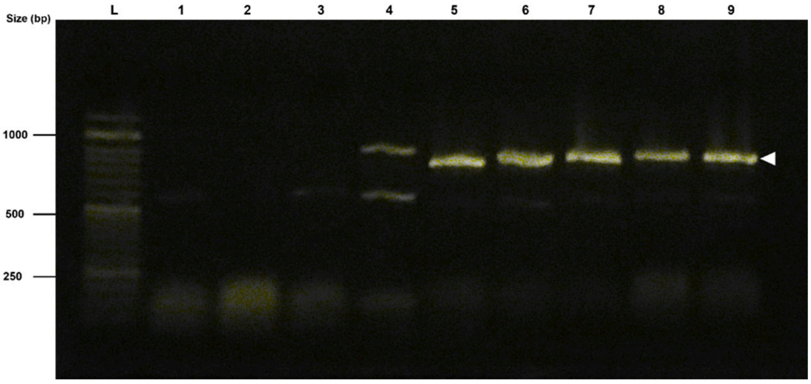

Figure S2. PCR screening of recombinant Pichia pastoris KM71 transformants. Agarose gel electrophoresis (1 %) of colony PCR products amplified using specific AOX primers. L: DNA molecular weight marker. Lanes 1–9: Randomly selected transformant colonies previously grown on histidine-deficient Minimal Dextrose (MD) solid medium. The white arrowhead indicates the expected ~750 bp amplicon, corresponding to the rBaMtx insert flanked by the AOX1 sequences. Positive clones showing a single, robust amplification band (e.g., lanes 5–9) were selected for subsequent heterologous expression.

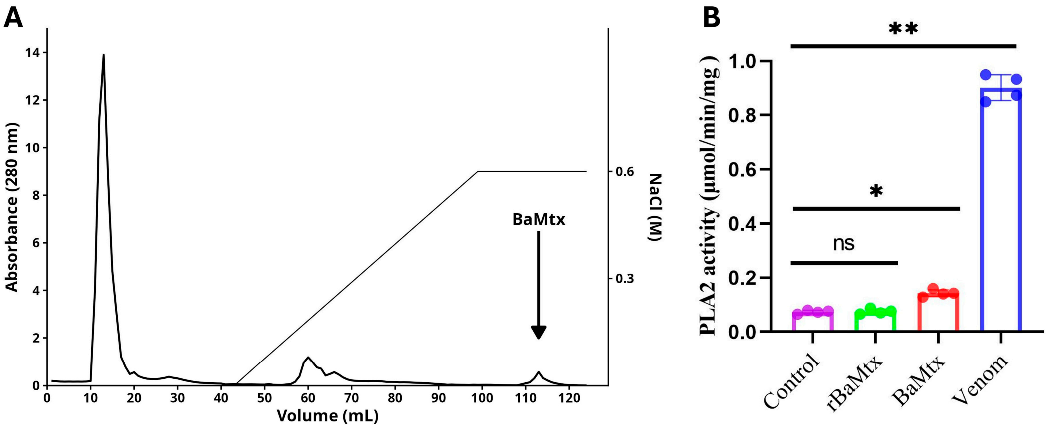

Figure S3. Chromatographic purification of native BaMtx and comparative PLA2 enzymatic activity. (A) Representative elution profile of crude B. atrox venom on a CM C-25 Sephadex cation-exchange column. The solid black line indicates the absorbance at 280 nm. Proteins were eluted using a linear gradient of 0 to 0.6 M NaCl (thin line) at a flow rate of 12 mL/min. The arrow indicates the peak corresponding to native BaMtx. (B) Specific PLA2 activity evaluated using the synthetic substrate NOBA. Assays were performed with PBS (negative control), rBaMtx, native BaMtx, and crude B. atrox venom. Data are expressed as mean ± SD (n = 4). Statistical significance was determined using One-way ANOVA followed by Tukey's test; *\$p < 0.01 and indicate significant differences compared to the PBS control; 'ns' indicates no significant difference.

Table S1. Cytokine concentrations (pg/mL) in 4T1 cell cultures treated with rBaMtx.

**Cytokines (pg/mL)**	**Concentration (μg/mL)**								**Detection limit (pg/mL)**
	100	50	25	5	1	0.5	0.1	Control	
**IL1- β**	18.6 ± 1.9	ND	ND	ND	ND	ND	ND	ND	8 – 1000
IL-6	ND	ND	ND	ND	ND	ND	ND	ND	4 – 500
TNF- α	ND	ND	ND	ND	ND	ND	ND	ND	8 – 1000
IL-10	ND	ND	ND	ND	ND	ND	ND	ND	32 – 4000

* Concentrations below the assay detection limit were considered not detectable (ND). Data are expressed as mean ± SD (n = 3). Control: RPMI.
